# Sodium channel myotonia may be associated with high-risk brief resolved unexplained events

**DOI:** 10.12688/wellcomeopenres.15798.2

**Published:** 2020-05-12

**Authors:** Gabriel Cea, Daniel Andreu, Elaine Fletcher, Sithara Ramdas, Richa Sud, Michael G. Hanna, Emma Matthews

**Affiliations:** 1 Departamento de Ciencias Neurológicas, Universidad de Chile, Santiago, Chile; 2 Servicio de Neurología, Hospital Salvador, Santiago, Chile; 3 Department of Clinical Genetics, Centre for Genomic and Experimental Medicine, Western General Hospital, Edinburgh, EH5 2GL, UK; 4 Department of Paediatric Neurology, John Radcliffe Hospital NHS Foundation Trust, Oxford, UK; 5 Neurogenetics Unit, UCL Queen Square Institute of Neurology, London, UK; 6 Department of Neuromuscular Diseases, UCL Queen Square Institute of Neurology, London, UK

**Keywords:** Sodium channel, Muscle Disease, Myotonia, Laryngospasm, Stridor, Apnoea, Channelopathy, Paediatric

## Abstract

Brief resolved unexplained events (BRUEs) have numerous and varied causes posing a challenge to investigation and management. A subset of infants with the neuromuscular disorder sodium channel myotonia, due to mutations in the *SCN4A* gene, experience apnoeic events due to laryngospasm (myotonia) of the upper airway muscles that may present as a BRUE. We sought to ascertain the frequency, severity and outcome of infants carrying the G1306E *SCN4A* mutation commonly associated with this presentation. We report 14 new cases of individuals with the G1306E mutation from three unrelated families and perform a literature review of all published cases. Infants with the G1306E mutation almost universally experience laryngospasm and apnoeic events. The severity varies significantly, spans both low and high-risk BRUE categories or can be more severe than criteria for a BRUE would allow. At least a third of cases require intensive care unit (ICU) care. Seizure disorder is a common erroneous diagnosis. Apnoeas are effectively reduced or abolished by appropriate treatment with anti-myotonic agents. Probands with the G1306E mutation who are family planning need to be counselled for the likelihood of post-natal complications. There is readily available and extremely effective treatment for the episodic laryngospasm and apnoea caused by this mutation. Proactively seeking clinical evidence of myotonia or muscle hypertrophy with consideration of CK,EMG and genetic testing in high risk BRUEs or more complex apnoeic events may reduce avoidable and prolonged ICU admissions, patient morbidity and potentially mortality.

## Introduction

Brief resolved unexplained events BRUEs (formerly apparent life threatening events, ALTEs) are defined by the American Academy of Pediatrics (AAP) as a sudden, brief (<1 min) resolved episode of one or more of: cyanosis or pallor, absent, decreased or irregular breathing, marked change in tone (hyper or hypotonia) and altered level of responsiveness occurring before the age of one year ^1^. The AAP have issued clinical guidelines on factors that allow a BRUE to be further classified as low risk or high risk ^1^. Low risk events are not thought to be associated with any increased risk of death or significant detrimental outcome and recommendations are provided to minimise unnecessary investigations or hospital admissions. High-risk events are more likely to be associated with an underlying disorder. Longer events or those with an abnormal history or examination at presentation are excluded from the definition of a BRUE and should prompt further investigation.

Mutations in the *SCN4A* gene cause a form of non-dystrophic myotonia known as paramyotonia congenita or sodium channel myotonia ^2^. The typical clinical presentation is of episodic myotonia (delayed muscle relaxation after contraction) affecting the limb and face muscles, exacerbated by exercise, cold temperature, potassium rich food, and associated with variable episodes of muscle weakness. Symptoms and morbidity can be significantly improved by the use of sodium channel blocking drugs e.g. mexiletine ^3, 4^.

A subset of infants, however, present with respiratory symptoms and/or laryngospasm due to myotonia of the respiratory and upper airway muscles ^5, 6^. The severity of these episodes varies from self-resolving in seconds to prolonged or recurrent events known as SNEL: severe neonatal episodic laryngospasm associated with hypertonia, apnoea, loss of consciousness and bradycardia ^7^. In between episodes, infants are usually well. Of the myotonic infants who present in this way the majority of them have carried the same *SCN4A* gene mutation, G1306E, p. (Gly1306Glu), usually in *De novo* form. Even in adults this mutation is regarded as somewhat of an outlier causing myotonia at the severe end of the spectrum, the description of symptoms caused by the mutation being termed “myotonia permanens” ^8^.

A clear understanding of the spectrum of severity and outcome is important to guide the specific monitoring and management of infants born with the G1306E mutation but there is a wider need to inform investigations for any infant presenting with recurrent apnoea or BRUE. We report 14 new cases from three unrelated families with the G1306E mutation and review the literature of all described cases to determine the extent, severity and treatment of myotonic symptoms and in particular respiratory complications.

## Methods

All procedures were conducted as part of routine clinical care.

### Standard protocol approval, registration and patient consent

The study was performed under the ethics guidelines issued by our institutions, with informed consent obtained from all participants for genetic studies and publication. We confirm that we have obtained permission to use images from the individuals included in this presentation, including explicit written permission from the mother to publish Figure 2.

Genetic analysis of the *SCN4A* gene was performed at the Neurogenetics Unit, National Hospital for Neurology and Neurosurgery as provided by the Channelopathy Highly Specialized National Service for rare disease. Samples underwent next-generation sequencing on an Illumina HiSeq after enrichment with an Illumina custom Nextera Rapid Capture panel (Illumina, Inc, San Diego, CA).

## Case reports

### Family A

**Figure 1.**
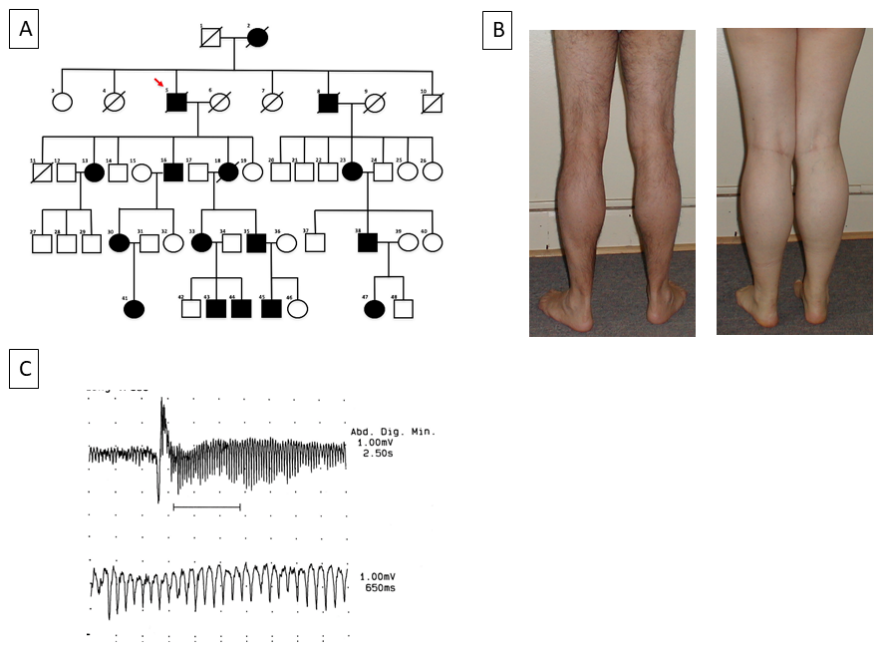
( **A**) Genogram of Family. Red arrow indicates index case. ( **B**) Calf hypertrophy in a male (case A:16) and female (case A:30). ( **C**) EMG showing myotonic activity exacerbated by needle movement from case A:30.

This large family from Chile includes 16 affected members of five generations who carry the *SCN4A* G1306E mutation ( Figure 1A). Detailed clinical history and examination were available for 12 individuals ( Table 1). All 12 had myotonia evident from birth, including laryngospasm and stridor that occurred universally in affected infants. During these episodes, the mothers reported that their babies stopped breathing for a few seconds, never more than a minute; they looked startled but did not become cyanosed. The episodes were more common in winter when they occurred one to two times a day on an almost daily basis, whereas in summer they occurred two to three times per week. They occurred mainly during the day but sometimes while the infants were sleeping. Cold environment, crying and breast or bottle-feeding were common provoking factors. These episodes were present until they were approximately 18 months old and the parents ultimately learnt to deal with them without seeking medical help. There was no history of prolonged respiratory complications requiring hospitalisation. In a few cases, salbutamol was given, as either an inhaler or syrup by their paediatricians, but this worsened the episodes to the point of cyanosis.

**Figure 2.**
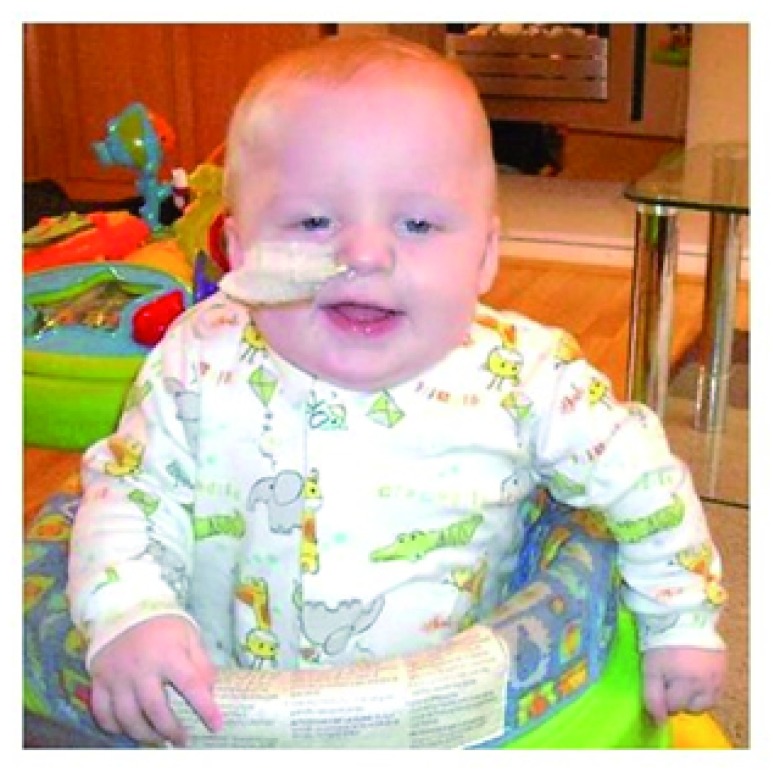
Case C as an infant demonstrating nasogastric tube required for feeding.

**Table 1.** Clinical features of new cases of G1306E.

Cases	A:5	A:16	A:30	A:41	A:18	A:33	A:43	A:44	A:35	A:45	A:13	A:23	B	C
**Current Age** **(years)**	Died at 86	60	37	9	Died at 69	42	11	10	34	7	61	79	36	9
**Age of onset of** **myotonia**	Birth	Birth	Birth	Birth	Birth	Birth	Birth	Birth	Birth	Birth	Birth	Birth	Infancy	5 days
**Neonatal** **Hypotonia**	No	No	No	No	No	No	No	No	No	No	No	No	No	No
**Inspiratory** **stridor/** **Laryngospasm**	Yes	Yes	Yes	Yes	Yes	Yes	Yes	Yes	Yes	Yes	Yes	Yes	Yes	Yes
**Myotonia** **distribution**	Generalized	Arms and legs	Generalized	Eyes, legs	Face, ocular, abdominal, arms, legs	Ocular, hands, legs	Ocular, hands, legs	Ocular, hands, legs	Ocular, hands, legs	Generalized	Generalized	Generalized	Hands, arms, legs, ocular	Generalised
**Site of severe** **myotonia**	Hands, legs	Hands	Hands, legs	Ocular, legs	Ocular, lhands, legs	Hands	Legs	Arms	Hands	Ocular	Ocular, legs and hands	Ocular, legs, arms	Hands, arms and legs	Ocular, Hands, Legs
**Exacerbation** **with cold**	(+)	(+)	(+)	(+)	(+)	(+)	(+)	(+)	(+)	(+)	(+)	(+)	(+)	(+)
**Exacerbation** **with exercise**	(+)	(+)	(+)	(+)	(+)	(+)	(-)	(-)	(+)	(+)	(+)	(+)	(+)	(-)
**Anaesthetic** **problems**	Yes	No	No	No	Yes	No	No	No	No	No	Yes	Yes	No	No
**Painful cramps**	Variable	Severe	Moderate	Moderate	Severe	Mild	Mild	Mild	Moderate	Mild	Moderate	Moderate	Moderate	Moderate
**Food Triggers**	Fish, Watermelon	Wine, watermelon cherry, banana, peach, fish, Tabaco	Wine, watermelon cherry, meat	Cherry, peach, watermelon, orange, fish	Fish, pork, meat, watermelon spinach	Fish, pork, meat, peach, cherry, banana, kiwi	Fish, pork, meat, cherry and peach	Fish, pork, meat, cherry and peach	Peach, cherry and banana	Fish, watermelon, pork, meat, cherry, peach	Watermelon, melon, banana,	melon, peach, grapes, apple, banana, tomato, fish, cucumber	None noted	None noted
**Muscle** **hypertrophy**	Calf	Calf	Calf	No	No	No	No	No	Calf	No	No	Calf	Calf, biceps, triceps	Trapezius, calf, biceps
**Creatine kinase** **IU/L**	568 */429 * 166	240	445/173	NA	552 * 290	260	NA	NA	240	NA	3300 ** 552 * 250	N/A * 330	N/A	

*Following adverse anaesthetic reaction. **Associated with influenza infection and respiratory failure. NA, not available.

All children had normal psychomotor development and no impairment in their activities of daily living. With increasing age, the myotonia was more generally distributed, particularly affecting the limbs and face (as is typical in older children and adults) and exacerbated by characteristic triggers of cold, physical activity, crying, emotional distress or potassium-rich food. Myotonia described as painful cramps was common in all cases and although these tended to be more frequent and incapacitating in females, they diminished in all affected family members, as they got older.

Examination demonstrated a robust physical build, more marked in the females than in the males ( Figure 1B). In all patients where creatine kinase (CK) level was assessed it was normal or elevated between two- to three-fold of normal values (24–170 U/L) in all cases but one (see Table 1). The exception was a patient whose CK level rose to 3300 U/L in the context of a severe respiratory insufficiency due to influenza infection, for which she was admitted to hospital. She was admitted to the intensive care unit (ICU) for 2 months on mechanical ventilation, due to a respiratory distress syndrome, secondary systemic infections and developed a critical illness neuropathy. She did not receive any specific treatment for her myotonia. Electromyography showed permanent myotonic discharges ( Figure 1C) in all cases.

In terms of treatment, most of the affected individuals chose not to take any medication as they felt symptoms were tolerable without. Eight had some benefit from using phenytoin or carbamazepine but took the medication sporadically due to side effects of feeling “floppy” and somnolent. Two were prescribed mexiletine but did not tolerate it due to a feeling of general malaise.

Other notable history was of adverse anaesthetic events in four family members ( Table 1) when erroneously given volatile induction anaesthetic and depolarizing agents (suxamethonium), consisting of moderate generalized myotonia including bulbar and jaw muscles that made intubation and ventilation difficult but not impossible. The episodes lasted for approximately 10 to 15 minutes and were not associated with any increase in body temperature, but in the three cases where CK level was available a moderate elevation was demonstrated (see Table 1). All deaths in family members were for causes unrelated to their myotonia.

### Proband B

A 36-year-old Caucasian woman was born by planned caesarean section at term in the U.K. with no concerns at birth. She was combination fed by breast and bottle without choking or swallowing difficulties.

During the first year of life, she was described as having “stiffening episodes” that lasted for seconds only and self-resolved; the main trigger was crying. On one occasion she was noted to be crying, became stiff, pale and appeared to lose consciousness, but recovered quickly. No other episodes were as dramatic as this and there were no admissions to hospital.

At around 18 months of age her mother first noted her defined leg and arm muscles whilst bathing her and parents observed her calves becoming “stiff” when she climbed stairs. A clinical diagnosis of myotonia congenita was made when the patient was approximately seven years old. Subsequent genetic investigations at age 30 years demonstrated the heterozygous pathogenic variant c.3917G>A, p. (Gly1306Glu), i.e. G1306E in the *SCN4A* gene confirming a diagnosis of sodium channel myotonia. Parents both tested negative for this variant and *De novo* inheritance was confirmed.

The main effects of her myotonia in adulthood are typical of sodium channel myotonia, e.g. myotonia of the arm and leg muscles causing difficulty closing/opening hands and impaired walking speed. These symptoms last for a few seconds, or minutes at most. Cold wind and exercise are exacerbating factors and symptoms are generally better in warm climates. Examination is notable for hypertrophy of calves, biceps, and triceps muscles. Acetazolamide (250 mg) twice daily gives good symptomatic control. Two previous uneventful anaesthetics have been given, one before her myotonic disorder was diagnosed, although we do not have details of the agents administered.

### Proband C

A nine-year-old non-identical male twin was born by emergency caesarean section in the U.K. for cord prolapse at 36+6 weeks gestation. He was healthy at birth with good APGAR scores. At 5 days old, he was admitted to his local hospital with recurrent episodes of apnoea, cyanosis and whole body “stiffening” that could occur up to 20 times a day. A seizure disorder was considered but EEG during these episodes was normal. His parents noted he had always had difficulty opening his eyes and choking on feeds with an unsafe swallow that required NG tube insertion ( Figure 2) and subsequently PEG tube insertion. At age 8 months examination demonstrated a Herculean appearance and eyelid myotonia. There was global developmental delay. There was no family history of note. EMG demonstrated myotonic discharges and genetic investigations confirmed a *De novo* *SCN4A* mutation c.3917G>A; p.G1306E. He was treated with phenytoin, carbamazepine and mexiletine prior to genetic diagnosis but without benefit. Episodes of laryngospasm significantly reduced with acetazolamide and he remains on this. He continues to have daily episodes of limb muscle myotonia which impair his motor abilities. He is now taking a normal oral diet and PEG tube was removed at 6 years of age. Episodes of laryngospasm resolved by the age of 7 years.

### Review of the literature

Literature review was conducted by searches of PubMed using a combination of the terms SCN4A, sodium channel myotonia, laryngospasm, stridor, and G1306E. Articles were also identified through searches of the authors own files. Only papers published in English were considered.

A review of the literature for the *SCN4A* G1306E mutation identified 30 affected individuals from 24 families. A summary of pertinent findings is summarised in Table 2 with a focus on laryngospasm, severity of respiratory complications, anaesthetic events and treatment.

**Table 2.** Summary of G1306E cases reported in the literature.

Case	Sex	Age at• onset of myotonia	Inheritance	Inspiratory stridor/ Laryngospasm	ICU care required	Anaesthetic reaction	Treatment	Ref
1	M	Birth	*De novo*	Yes	Yes –mask ventilation and oxygen therapy		NS	9
2	F	Birth	*De novo*	Yes			Mexiletine	9
3	F	Neonate	*De novo*	Yes			Carbamazepine and mexiletine effective	9
4	F	Birth	*De novo*. Mother of 4:1	Yes	NS but LOC reported and recurrent hospitalisation		Flecainide more effective than carbamazepine or mexiletine	9, 11
4:1	M	Birth	AD Son of 4	Yes			Mexiletine – limited benefit Flecainide much more effective	9, 11
5	M	Birth	*De novo*	Yes	NS but LOC reported and recurrent hospitalisation		Mexiletine better than carbamazepine	9
6	F	Infant	*De novo*	Yes			Flecainide effective Mexiletine + lamotrigine –not effective	9
7	M	Neonate	*De novo*	Yes	NS but LOC reported and recurrent hospitalisation		Mexiletine	9
8	M	Neonate	*De novo*	No			Mexiletine	9
9 *	M	Neonate	Reported *De novo*	Yes	NS but LOC reported and recurrent hospitalisation	Yes – fatal MH like event	Dilantin, desipramine	9
9:1	M	Birth		Onset at 4 months			Dilantin, desipramine	9
9:2	F	Neonate		Yes		Yes succinylcholine induced event	Dilantin, despramine	9
10 **	M	Birth	*De novo*	Onset at 9 months			Mexiletine	9
11	M	Early childhood	AD	No (some bulbar and respiratory symptoms reported but not severe)	No		Mexiletine, Oxcarbazepine, thiazide diuretics – no benefit	
Acetazolamide – some improvement	15						
11:1	M	Birth	AD Son of 11	No	No		None	12
12	M	Birth	*De novo*	No	No		None reported	13
13	M	Childhood	Presumed *De novo*	No	No		None reported	13
14	F	Birth	*De novo*	Yes	NS but recurrent hospitalisation		Carbamazepine – minimal benefit Mexiletine + Acetazolamide –minimal benefit Flecainide – good response	7
15	M	Neonate	*De novo* (+CLCN1 M485V)	Yes	No		Mexiletine – good response	14
15:1+ 15:2	M	Neonate	AD (G1306E +M485V) Monozygotic twin sons of 15	Yes	no		No treatment	14
16	M	Neonate	*De novo*	Yes	Yes – tracheostomy 3months		Carbamazepine – good response, tracheostomy removed	6
17	M	Neonate	*De novo*	yes	Yes		Mexiletine – good response	6
18	F	Birth	NS	Yes	NS		NS	15
19	M	Birth	*De novo*	Yes	Yes		Mexiletine – some benefit Further benefit from addition of acetazolamide	10
20	M	Birth	*De novo*	Yes	Yes		Carbamazepine – good response	10
21	F	8 weeks	*De novo*	Yes	Yes		Carbamazepine – good response	16
16	F	Neonate	NS – father possibly affected	Yes	No		Carbamazepine – good response	16
23	F	Neonate	AD – father affected	Yes	No		Carbamazepine – good response	16
24	M	Neonate	*De novo*	Yes	NS		Mexiletine - good response, abolished respiratory episodes	17

* Died of cardiorespiratory failure age 11 following General anaesthetic with Malignant Hyperthermia (MH) like event (severe myotonia) and hyperkalaemia.

** Died age 22 – cause unclear. NS, not specified. AD: autosomal dominant, LOC: loss of consciousness.

**Figure 3.**
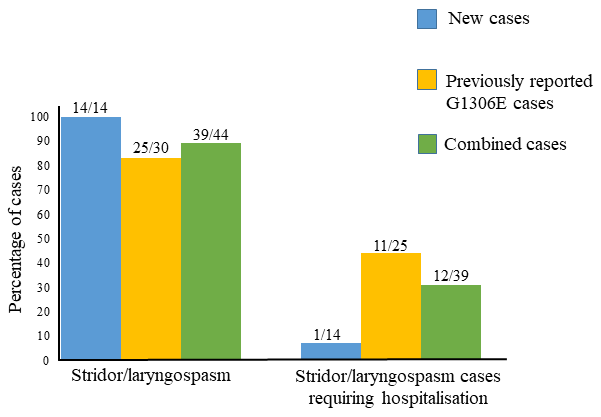
Frequency of G1306E cases experiencing stridor/laryngospasm and the requirement for hospitalisation.

The literature review mirrored the frequency of stridor/laryngospasm and respiratory events seen in our newly described patients (100%) with 25/30 (83%) reported G1306E cases experiencing these symptoms ( Figure 3). Similar to our cases, this also began at birth or in the neonatal period in the majority although onset could be delayed by up to nine months of age ( Table 2). A total of 11/25 (46%) of those experiencing stridor/laryngospasm who were reported in the literature had data available to demonstrate they required recurrent hospitalisation or ICU care for respiratory compromise which could be up to 6 months in duration before an accurate diagnosis was made. In every case requiring hospitalisation, the G1306E mutation had arisen *De novo* in the child. This may reflect more severe myotonia associated with de novo mutation or that in cases where parents were themselves affected they were more aware of the risk of myotonia including respiratory presentations and better prepared to manage these symptoms.

Nearly all reported cases received pharmacological treatment. The effect of treatment on laryngospasm was often dramatic and rapid with complete resolution or significant improvement of apnoeas enabling discharge from hospital within days. Adverse anaesthetic events were rare (2 cases), but fatal in one case. In both, suxamethonium had been administered ^9^.

## Discussion and conclusions

Myotonia caused by mutations in the *SCN4A* gene was generally considered debilitating but not a life-threatening condition. More recently, the severe laryngeal and respiratory muscle presentations seen in infancy have coined the phenotype SNEL (severe neonatal episodic laryngospasm) ^6, 10^. In between episodes, infants usually appear well and many have been misdiagnosed with reflux, laryngomalacia or epilepsy (despite a normal EEG) ^9^. Substantial delay in diagnosis is common with the more severe cases spending on average 6 months in ICU ^5, 6, 9^. The condition is, however, very sensitive to treatment with anti-myotonic agents, usually sodium channel blockers, which have a dramatic effect on apnoeic symptoms ( Table 2).

Although several mutations have been reported to cause this phenotype, the G1306E mutation occurs in the majority of affected individuals, frequently in *De novo* form.

We report the largest single family described with the G1306E mutation and two unrelated *De novo* cases. Our cases and literature review highlight several key variables. Stridor and laryngospasm from birth are almost universal but the severity of these episodes varies considerably from a single self-resolving event to to the typical SNEL phenotype with more than 20 episodes a day, prompting hospitalisation and ICU admission. If our families are combined with current literature, 31% of infants with stridor/laryngospasm require hospital intervention compared to 44% based on the literature search alone ( Figure 3). Bias towards under-representing the frequency of these events may be introduced by our single large family A who were relatively mildly affected but conversely bias towards over-representing them may come from a tendency for more severe cases to be reported in the literature.

Infants with the G1306E mutation are likely to present initially to general physicians and departments with the shorter, self-resolving events potentially falling within the definition of a BRUE. In the majority these would be classified as a high-risk BRUE due to the recurrent nature of events ^1^. Some of the children (those fitting the SNEL phenotype), had episodes longer than one minute and/or more complicated histories, which would exclude them from the BRUE classification. Abnormal examination is also not consistent with a BRUE e.g. Proband C who demonstrated global developmental delay, but the typical signs of a myotonic disorder can be delayed e.g. muscle hypertrophy may not be present before age 6 months or difficult to detect in a general setting. High-risk BRUEs and longer episodes with abnormal history or examination (i.e. not a BRUE) should prompt further investigations but it is notable that no recommendations exist as to what investigations should be performed or standardised ^1, 18^.

Sodium channel myotonia is a skeletal muscle exclusive presentation. It is not feasible or suggested that CK and/or EMG be performed in every infant presenting with a possible BRUE. However, they can be helpful in the more severe cases of SNEL where delayed diagnosis is common, resulting in unnecessary and prolonged ICU stays and preventable death ^10^. CK is a non-specific test which can be raised in non-neuromuscular disease e.g. post seizure and seizure disorder is a common differential diagnosis postulated in SNEL cases ^19^. A normal EEG, however, should prompt consideration of a peripheral rather than central disorder. EMG demonstrating myotonia is much more indicative of the diagnosis but can be difficult to perform and interpret in young infants or may not be available outside of specialist centres. Positive results can be useful for indicating genetic analysis of the *SCN4A* gene and guiding the interpretation of any gene variants. Despite the potential limitations of investigations, there is readily available and extremely effective treatment for the laryngospasm associated with sodium channel myotonia, making it essential that this differential diagnosis and genetic testing where available, is considered in all infants presenting with a high-risk BRUE or more complex apnoeic episodes.

The mainstay of treatment for sodium channel myotonia is sodium channel blocking drugs and our literature review demonstrates efficacy from mexiletine, carbamazepine and flecainide. There is in vitro evidence that flecainide may be the the most potent treatment for the G1306E mutation ^11^ but there are too few clinical cases and no clinical trial to draw firm conclusions over superiority.

We recently reported *SCN4A* mutations with qualitatively similar effects on channel function in a cohort of infants who had died from sudden infant death ^20^. It is generally considered that infants who present with low-risk BRUEs are not at any greater risk of sudden infant death but data are unclear for high-risk BRUEs ^14^. No cases of sudden or any infantile deaths were reported in our large autosomal dominant family although their clinical severity was much less than many of the *De novo* cases. Two infants who presented with laryngospasm due to other *SCN4A* mutations have died from respiratory complications ^5, 10^. The association of *SCN4A* variants and sudden infant death warrants further evaluation.

Other notable differences between our family A and the majority of cases in the literature were the lack of requirement for medication and less incapacitating limb myotonia experienced in adulthood. One other autosomal dominant family previously reported to highlight a milder phenotype is also of Chilean origin ^12^. To our family’s knowledge, these individuals are not directly related to them but whether other genetic variants common to their ethnicity could influence the severity of clinical symptoms is unclear.

Anaesthetic agents especially suxamethonium are known to exacerbate myotonia, potentially making intubation impossible as well as causing hyperkalaemia ^22, 23^. Hyperkalaemia itself is cardiotoxic but in the case of *SCN4A* related myotonia is also known to exacerbate myotonic symptoms. Our case series and literature review have highlighted the gravitas of an erroneous administration of suxamethonium including fatal outcome ^9^. We would emphasise that suxamethonium use be considered contraindicated in *SCN4A* related myotonia and periodic paralysis.

In summary, episodic laryngospasm and stridor typically from birth are almost universal symptoms in those with the S *CN4A* G1306E mutation and affected parents should be counselled for this when family planning. We would recommend labour take place in a hospital with senior paediatric staff and ICU facilities available. Subsequent respiratory complications during infancy are common but vary significantly in severity. An enhanced awareness of the possibility of sodium channel myotonia, with consideration of CK plus/minus EMG and genetic analysis in appropriate cases of high-risk BRUEs and more complex recurrent apnoeas could enhance diagnostic rates, reducing morbidity and potentially mortality.

## Data availability

All data underlying the results are available as part of the article and no additional source data are required.

## Consent

Written informed consent for publication of the participants’ details and their images was obtained from the participants or parents/guardians of the participants.
