# Supplementary material for: Sodium channel myotonia may be associated with high-risk brief resolved unexplained events
Source: Wellcome Open Res. 2020 May 12;5:57. Originally published 2020 Mar 31. [Version 2] doi: 10.12688/wellcomeopenres.15798.2 (PMC7241273; doi:10.12688/wellcomeopenres.15798.2)
Supplement: Peer review history

# Peer review: Reviewer response for version 2

**DOI:** 10.21956/wellcomeopenres.17445.r38700

**Published:** 2020-05-20

**Authors:** Sophie Nicole

**Affiliations:** 1 Institut génomique fonctionnelle, UM, Inserm, CNRS, Paris, France

I have no further comments, the answers and manuscript changes made by the authors being convincing.

Are enough details provided of any physical examination and diagnostic tests, treatment given and outcomes?

Partly

Is the background of the cases’ history and progression described in sufficient detail?

Yes

Is sufficient discussion included of the importance of the findings and their relevance to future understanding of disease processes, diagnosis or treatment?

Partly

Is the conclusion balanced and justified on the basis of the findings?

Yes

Reviewer Expertise:

Neurogenetics, physiology of muscle sodium channel disorders but not medicine

I confirm that I have read this submission and believe that I have an appropriate level of expertise to confirm that it is of an acceptable scientific standard.

# Peer review: Reviewer response for version 2

**DOI:** 10.21956/wellcomeopenres.17445.r38701

**Published:** 2020-05-13

**Authors:** Maria Elena Farrugia

**Affiliations:** 1 Department of Neurology, Institute of Neurological Sciences, Queen Elizabeth University Hospital, Glasgow, UK

I am happy that all points were addressed effectively.

Are enough details provided of any physical examination and diagnostic tests, treatment given and outcomes?

Yes

Is the background of the cases’ history and progression described in sufficient detail?

Yes

Is sufficient discussion included of the importance of the findings and their relevance to future understanding of disease processes, diagnosis or treatment?

Yes

Is the conclusion balanced and justified on the basis of the findings?

Yes

Reviewer Expertise:

NA

I confirm that I have read this submission and believe that I have an appropriate level of expertise to confirm that it is of an acceptable scientific standard.

# Peer review: Reviewer response for version 1

**DOI:** 10.21956/wellcomeopenres.17324.r38298

**Published:** 2020-04-27

**Authors:** Maria Elena Farrugia

**Affiliations:** 1 Department of Neurology, Institute of Neurological Sciences, Queen Elizabeth University Hospital, Glasgow, UK

This is a very well written manuscript describing the phenotype of 12 new cases of individuals with sodium channel myotonia with the G1306E variant and with comparison with published cases in the literature. I have the following, relatively minor, points:

1. In my opinion the authors should mention SNEL in the introduction and this should flow from BRUE, highlighting the differences between the 2. There is the danger of the 2 terms being used interchangeably so differences in the duration of events in the 2 entities, prognostic differences and what determines how a physician classifies an event as SNEL versus BRUE would be helpful in the introduction of the manuscript. The mention on SNEL in the first paragraph of the discussion seems a bit misplaced.
2. Do we know from in vitro studies what determines an individual's response to a specific sodium channel blocker? It would be worth including this in discussion if known. I was interested that Lamotrigine was not tried in any of the cases. Is this simply coincidence?
3. Do we know why the episodes of layngospasm tend to resolve after childhood? Are there factors in the embryology, development, maturation of laryngeal muscles that make them more susceptible in infancy? It would be interesting to hypothesise in the discussion.
4. In table 1 the plus sign under C for "exacerbation with cold" has no bracket. Does this denote something different to the rest (with brackets)? I suspect not but specify if it is.
5. The title of Table 2 is clearly erroneous. Please amend. I would also suggest denoting the abbreviations used in table 2 even though they are obvious to most.

Are enough details provided of any physical examination and diagnostic tests, treatment given and outcomes?

Yes

Is the background of the cases’ history and progression described in sufficient detail?

Yes

Is sufficient discussion included of the importance of the findings and their relevance to future understanding of disease processes, diagnosis or treatment?

Yes

Is the conclusion balanced and justified on the basis of the findings?

Yes

Reviewer Expertise:

I do very little research since nonacademic but mostly in areas of myasthenia gravis, myotonic dystrophy, fatigue in neuromuscular disorders. However, I review many manuscripts, grant and book proposals.

I confirm that I have read this submission and believe that I have an appropriate level of expertise to confirm that it is of an acceptable scientific standard.

# Response

**Published:** 2020-05-01

**Authors:** Emma Matthews

**Affiliations:** University College London, UK

This is a very well written manuscript describing the phenotype of 12 new cases of individuals with sodium channel myotonia with the G1306E variant and with comparison with published cases in the literature. I have the following, relatively minor, points:

 1. In my opinion the authors should mention SNEL in the introduction and this should flow from BRUE, highlighting the differences between the 2. There is the danger of the 2 terms being used interchangeably so differences in the duration of events in the 2 entities, prognostic differences and what determines how a physician classifies an event as SNEL versus BRUE would be helpful in the introduction of the manuscript. The mention on SNEL in the first paragraph of the discussion seems a bit misplaced.

 The reviewer makes a good point and the introduction and explanation of the term SNEL earlier in the manuscript was also suggested by another reviewer. We have done this now in the abstract and introduction.

 2. Do we know from in vitro studies what determines an individual's response to a specific sodium channel blocker? It would be worth including this in discussion if known. I was interested that Lamotrigine was not tried in any of the cases. Is this simply coincidence?

 We think lamotrigine may not have been tried as it has only relatively recently been identified as an effective anti-myotonic and many of the G1306E cases in the literature predate this. There is some in vitro evidence that of different sodium channel blockers flecainide may be the most efficacious for the G1306E mutation but there are no clinical trials comparing different agents yet to establish this. We have added this point to the discussion.

 3. Do we know why the episodes of layngospasm tend to resolve after childhood? Are there factors in the embryology, development, maturation of laryngeal muscles that make them more susceptible in infancy? It would be interesting to hypothesise in the discussion.

 We don’t know why but we agree with the reviewer that the resolution with age implies a developmental factor(s). We hypothesise that the relative proportion of fast and slow twitch muscle fibres in different muscles and the change in these proportions with age may be one of these developmental factors but at present this is our hypothesis only. As it is a speculative notion, requiring further research, we prefer not to include this to avoid any confusion.

 4. In table 1 the plus sign under C for "exacerbation with cold" has no bracket. Does this denote something different to the rest (with brackets)? I suspect not but specify if it is.

 No there was no reason for this and we have added brackets for consistency.

 5. The title of Table 2 is clearly erroneous. Please amend. I would also suggest denoting the abbreviations used in table 2 even though they are obvious to most.

 We have amended this and detailed the abbreviations.

# Peer review: Reviewer response for version 1

**DOI:** 10.21956/wellcomeopenres.17324.r38296

**Published:** 2020-04-07

**Authors:** Sophie Nicole

**Affiliations:** 1 Institut génomique fonctionnelle, UM, Inserm, CNRS, Paris, France

This manuscript reports the clinics of 12 patients (3 unrelated families) with brief resolved unexplained events in their neonatal period related to sodium channel myotonia and one missense mutation (G1306E) in the *SCN4A *gene. A review of the literature is done to compare the clinics with 30 patients (24 families) with this phenotype and *SCN4A* mutation already described in the literature. This disease is ultra-rare and deserves to be well described in the literature as it may be confounded with seizure disorder in neonates. However, it is a treatable disease with sodium channel blockers, has a good prognosis and may benefit from genetic counseling so well communicating on it is important.

 The manuscript is well written and greatly increase the total number of patients description. To my opinion, referring to Severe Neonatal Episodic Laryngospasm (SNEL) earlier in the manuscript would help the readers not familiar with sodium channel myotonia (SCM) to get the point. The authors never use this term of “Severe Neonatal Episodic Laryngospasm (SNEL)” in the title, the abstract, the introduction or the keywords although the SNEL term is used by distinct authors in the literature and is known to be associated with ( *de novo*) G1306E mutation of *SCN4A *(as indicated by the authors in the discussion). Could these episodes be really classified as “unexplained” as they are the clinical signs of neonatal SCM caused by one *SCN4A* missense mutation? I recommend the authors to best underline (title, abstract, introduction, discussion) the fact that some BRUE may be indeed “SNEL” related to SCM with efficient treatment and good prognosis. They may also more clearly discuss on the fact that any *de novo* SNEL form may become inherited (as well illustrated by family A), and so may benefit from genetic counselling.

 The authors recommend to search for clinical evidence of myotonia/muscle hypertrophy with consideration of CK and EMG in case of BRUE. Why do they not recommend to search for *SCN4A* mutations, at least the few ones (including G1306E) known to cause SNEL?

 Regarding Family A, 10 individuals are indicated as having available clinical elements in the text but 12 are described in table 1. How was the SCM diagnosis establish for individual A2 ( *SCN4A* sequencing? Clinical report?)? Regarding the critical point which is the treatment, could the authors be more precise: how many exactly benefited from using phenytoin or carbamazepine? What were the adverse events with mexiletine? In table 1, myotonia distribution in eyes: did it mean ocular, orbital, palpebral? What is the difference between mild and moderate painful cramps? At which age was noticed muscle hypertrophy (birth? childhood? adulthood?)? What was the frequency of laryngospasm episodes?

 Regarding the review of the literature, the authors indicated that all hospitalization cases were associated with *de novo* mutation. Could they further discuss about the, at least, two distinct possibilities to explain this observation, which are: 1) that may be due to the more severe SCM related to G1306E or 2) that may be due to the fact that, as one of the parents was affected by SCM, they were aware of the SNEL risk and so known how to prevent / best manage it?

 The title of Table 2 is strange (and probably wrong). What do AD, LOC, GA, MH mean? How was estimated the treatment efficiency (EVA? PRO? EMG?).

Are enough details provided of any physical examination and diagnostic tests, treatment given and outcomes?

Partly

Is the background of the cases’ history and progression described in sufficient detail?

Yes

Is sufficient discussion included of the importance of the findings and their relevance to future understanding of disease processes, diagnosis or treatment?

Partly

Is the conclusion balanced and justified on the basis of the findings?

Yes

Reviewer Expertise:

Neurogenetics, physiology of muscle sodium channel disorders but not medicine

I confirm that I have read this submission and believe that I have an appropriate level of expertise to confirm that it is of an acceptable scientific standard, however I have significant reservations, as outlined above.

# Response

**Published:** 2020-04-24

**Authors:** Emma Matthews

**Affiliations:** University College London, UK

This manuscript reports the clinics of 12 patients (3 unrelated families) with brief resolved unexplained events in their neonatal period related to sodium channel myotonia and one missense mutation (G1306E) in the SCN4A gene. A review of the literature is done to compare the clinics with 30 patients (24 families) with this phenotype and SCN4A mutation already described in the literature. This disease is ultra-rare and deserves to be well described in the literature as it may be confounded with seizure disorder in neonates. However, it is a treatable disease with sodium channel blockers, has a good prognosis and may benefit from genetic counseling so well communicating on it is important.

The manuscript is well written and greatly increase the total number of patients description. To my opinion, referring to Severe Neonatal Episodic Laryngospasm (SNEL) earlier in the manuscript would help the readers not familiar with sodium channel myotonia (SCM) to get the point. The authors never use this term of “Severe Neonatal Episodic Laryngospasm (SNEL)” in the title, the abstract, the introduction or the keywords although the SNEL term is used by distinct authors in the literature and is known to be associated with (de novo) G1306E mutation of SCN4A (as indicated by the authors in the discussion). Could these episodes be really classified as “unexplained” as they are the clinical signs of neonatal SCM caused by one SCN4A missense mutation? I recommend the authors to best underline (title, abstract, introduction, discussion) the fact that some BRUE may be indeed “SNEL” related to SCM with efficient treatment and good prognosis.

The authors agree with the reviewer that the term SNEL does refer specifically to the severe episodes of laryngospasm, stridor and respiratory complications associated with sodium channel myotonia. In this manuscript we have sought to illustrate that infants with the G1306E mutation (along with those with other SCN4A mutations) experience myotonia of the laryngeal and respiratory muscles that cause episodic events of variable severity. This ranges from a single relatively mild self-resolving episode lasting less than a minute through to the much more severe typical SNEL episodes. We use the term BRUE as this is more likely to be the initial category used (before diagnosis) to describe many of these cases, especially the milder ones when they first present to paediatricians who are not neuromuscular specialists. To address the reviewer’s point we have edited the introduction to include the term SNEL earlier with reference to the severe SCN4A cases and specified it again in the first paragraph of discussion.

They may also more clearly discuss on the fact that any de novo SNEL form may become inherited (as well illustrated by family A), and so may benefit from genetic counselling.

The reviewer is correct that de novo cases may then have affected children of their own as illustrated in Family A and Table 2. We agree this is important to highlight and our summary includes the recommendation that any affected individual who is family planning be counselled for the risks of having an affected child.

The authors recommend to search for clinical evidence of myotonia/muscle hypertrophy with consideration of CK and EMG in case of BRUE. Why do they not recommend to search for SCN4A mutations, at least the few ones (including G1306E) known to cause SNEL?

We agree this is important and will ultimately give a definitive diagnosis although genetic testing can still be relatively time consuming (in comparison to EMG and CK), costly and not readily available in all countries. Results should ideally be interpreted alongside clinical, EMG and CK data as without this variants of uncertain significance may cause uncertainty and diagnostic confusion. We have revised the text in the abstract, discussion and summary to include consideration of genetic analysis guided by clinical presentation and results of these other investigations.

Regarding Family A, 10 individuals are indicated as having available clinical elements in the text but 12 are described in table 1.

This was an error and we are very grateful to the reviewer for pointing this out. We have amended the text throughout and values in figure 1 accordingly.

How was the SCM diagnosis establish for individual A2 (SCN4A sequencing? Clinical report?)?

Diagnosis was based on clinical report of several family members. 

Regarding the critical point which is the treatment, could the authors be more precise: how many exactly benefited from using phenytoin or carbamazepine?

Seven patients used carbamazepine: A 13, A 16, A 35, A 43, A 44, A 45). They feel that myotonia and cramps improved but their use is sporadic because they feel floppy and somnolent when taking carbamazepine and they do not like that state. One patient used phenytoin: patient (A 23).

What were the adverse events with mexiletine?

It was taken by only two family members (patients A23, A47) as it was not available for a long time in Chile. A23 stated that she felt shaky and generally unwell while taking mexiletine.

We have added additional detail to the text regarding the use and outcome of these medications.

In table 1, myotonia distribution in eyes: did it mean ocular, orbital, palpebral?

Ocular involvement included orbicularis oculi (tonic eyelid closing) and extrinsic ocular muscle spasm (tonic gaze). The latter occurs with quick changes of gaze or when they yawn, the eye gets fixed in one position and may take a few seconds before the patient can move their eye normally. Sometimes they have diplopia during the episode.  These were described by patients:  A 18, A 23, A 30, A 33, A 35, A 41, A 43, A 45, A 44.

Only one patient experienced tonic eyes opening A33 (eyelid levator muscle).

What is the difference between mild and moderate painful cramps?

It is a clinical assessment. Mild cramps do not stop  daily living activities; with moderate cramps, the patient experiences some pain and some interference with activities of daily living, sometimes having to stop what they are doing for a few seconds; Severe cramps lasts for some minutes or hours: “I become a robot” as they describe it. Severe cramps prevent some activities of daily living and the patient experiences more intense pain, especially when using manoeuvers like stretching or moving to try to stop the myotonia.

At which age was noticed muscle hypertrophy (birth? childhood? adulthood?)?

It is very difficult to establish a starting point with any certainty. Parents say that all their children have important muscular bulk from birth but, if they have to mention a time when it became really evident, most say after 2 years of age.

What was the frequency of laryngospasm episodes?

Please see the text describing this family in the first paragraph of Family A, line 9: “the episodes were more common in winter when they occurred one to two times a day on an almost daily basis, whereas in summer they occurred two to three times per week”.

Regarding the review of the literature, the authors indicated that all hospitalization cases were associated with de novo mutation. Could they further discuss about the, at least, two distinct possibilities to explain this observation, which are: 1) that may be due to the more severe SCM related to G1306E or 2) that may be due to the fact that, as one of the parents was affected by SCM, they were aware of the SNEL risk and so known how to prevent / best manage it?

We agree and have included discussion in the text.

The title of Table 2 is strange (and probably wrong). What do AD, LOC, GA, MH mean? How was estimated the treatment efficiency (EVA? PRO? EMG?).

The title is wrong, and we think is from a different publication substituted in the publishing process, but we have amended this to the correct title for Table 2. We have detailed the meaning of the acronyms.

Treatment efficacy had to be surmised from the detail provided in the reported publications and was described in different ways.
